# Towards a forward-looking ethnobiology: envisioning and co-creating biocultural futures

**DOI:** 10.1186/s13002-025-00820-1

**Published:** 2025-10-27

**Authors:** Álvaro Fernández-Llamazares, Irene Teixidor-Toneu

**Affiliations:** 1 Departament de Biologia Animal, Biologia Vegetal i Ecologia (BABVE), Universitat Autònoma de Barcelona, Cerdanyola del Vallès, Barcelona, Spain; 2 Institut de Ciència i Tecnologia Ambientals (ICTA‑UAB), Universitat Autònoma de Barcelona, Cerdanyola del Vallès, Barcelona, Spain; 3 IMBE, Aix Marseille Univ, Avignon Univ, CNRS, Marseille, IRD France

**Keywords:** Biocultural resilience, Futures thinking, Participatory action, Transformative change

## Abstract

In the face of accelerating environmental and socio-political changes, there is value in expanding the temporal scope of ethnobiology to more actively engage with the future. This perspective explores the potential of a forward-looking ethnobiology that incorporates methods from Futures Studies to co-envision and co-produce sustainable biocultural futures in partnership with Indigenous Peoples and local communities. We highlight different methods and tools that can be repurposed to create inclusive, transdisciplinary spaces for community-led imagination, experimentation, and learning. By embedding futures thinking into the fabric of ethnobiological practice, the discipline can further enrich its longstanding role in fostering biocultural resilience. We argue that the time has come not only to imagine the future of ethnobiology, but to actively co-create it through culturally grounded, future-oriented, and ethically engaged methodologies. This shift repositions ethnobiology as a central force in advancing just and sustainable pathways.

## Introduction

Ethnobiology has traditionally focused on documenting the dynamic relationships between people and their environments, with a strong emphasis on historical continuity, cultural memory, and the present-day relevance of ethnobiological knowledge [1, 2]. Ethnobiologists regularly take the pulse of our joint academic efforts and look forward, identifying emerging directions in need for collective attention (e.g. [3–5]). Among the various visions for ethnobiology, one of the most recurrent is the call for the discipline to play a more active role in addressing environmental and social injustices (e.g. [6–12]). In the twenty-first century, more than ever, interlinkages between societies and the environment operate across multiple spatial and temporal scales, making these intertwined dynamics increasingly significant, complex and unpredictable [13, 14]. Building on the principles set forth in the Declaration of Belém [15], ethnobiologists should continue to stand alongside Indigenous Peoples, local communities, traditional peoples, and Afro-descendant groups to better understand global change and respond to the complex, interwoven challenges threatening livelihoods and planetary well-being. In this piece, we highlight emerging opportunities to advance this decades-old call for meaningful engagement in addressing global crises. We propose to do so by expanding the temporal scope of the discipline to engage more directly with the future [16].

This does not imply a redirection of ethnobiology’s core analytical focus, but rather an expansion of its temporal and methodological horizons. The meticulous documentation of ethnobiological knowledge, which is often grounded in historical depth and cultural continuity, remains a cornerstone of the discipline and a vital contribution to both scholarship and practice [17–19]. Our intention here is not to replace this foundational work, but rather to complement it with forward-looking approaches that can support communities in navigating contemporary and future challenges.

A forward-looking ethnobiology should not only hypothesize on what lies ahead, but more importantly, actively collaborate with Indigenous and local in co-envisioning desirable futures, identifying risks and opportunities, and informing both policy and practice. This shift requires a rethinking of ethnobiology’s role not only as a chronicler of cultural and ecological memory, but also as a field engaged in imagining and facilitating innovative responses to emerging challenges [2, 9, 7]. Theoretically, our perspective aligns with emerging frameworks of environmental and ancestral futurism (e.g. [20, 21]), which centre long-term ecological care and intergenerational responsibilities within futures-oriented thinking. Building on this theoretical body, we explore how a range of participatory methods and approaches might serve as catalysts for inclusive, community-driven processes of envisioning and shaping more just and sustainable biocultural futures.

By “bringing the past forward into the future” [22], ethnobiology can position itself as a critical actor in shaping resilient and equitable pathways in an increasingly uncertain world [8], [23, 11]. This forward-looking orientation is not entirely new. The International Society of Ethnobiology’s Declaration of Belém [15] already articulated intergenerational responsibilities for sustaining biocultural diversity. Since then, scenario planning has been applied in different ethnobiological contexts to anticipate different social-ecological impacts and co-design adaptive strategies [24–26]. Ethnobiological knowledge has also been increasingly mobilized in discussions of sustainability transitions [27, 2], while research on agrobiodiversity and traditional food systems has contributed to seed sovereignty and biocultural resilience [28–31]. Taken together, these pioneering contributions demonstrate that ethnobiology has long engaged in anticipatory and future-oriented practices. Our aim is to build on and connect this rich body of work to the conceptual and methodological repertoire of Futures Studies.

Futures Studies is an interdisciplinary field concerned with the systematic exploration of possible, probable, and preferable futures, and the worldviews and values that underlie them [32]. Emerging in the mid-twentieth century, Futures Studies draws from methods such as scenario planning, visioning, horizon scanning, and back-casting, with the aim of supporting more informed decision-making and adaptive capacity in the face of uncertainty [33, 34]. While other envisioning approaches (e.g. participatory scenario planning, environmental foresight, and anticipatory governance) share overlapping goals, Futures Studies is distinctive in its systematic orientation towards multiple futures, its focus on both exploratory and normative dimensions, and its strong emphasis on plural, inclusive perspectives [35]. Forward-looking ethnobiology complements and extends these traditions by grounding futures thinking in place-based knowledge systems, cultural values, and stewardship practices.

Integrating approaches from Futures Studies offers a promising avenue to broaden both the epistemological and methodological horizons of ethnobiology. Ethnobiologists can draw inspiration from place-based futures thinking, an emerging family of participatory methods and scenario-building exercises that underscore the role of envisioning positive futures in building transformative capacity [36, 37]. Futures thinking encourages the exploration of multiple possible, probable, and desirable futures [38], while scenario planning provides a structured framework for exploring plausible trajectories amid uncertainty [24, 39, 40, 25]. These approaches are particularly well suited to engage with Indigenous and local knowledge systems, which often hold deeply embedded anticipatory logics and long-term ecological foresight [41–43].

## Methodological gateways to ethnobiological futures

Embedding such future-oriented methods within ethnobiology can further enrich the discipline’s longstanding commitment to applied, anticipatory, and co-creative work, helping to advance towards sustainable biocultural futures [44]. Methodologically, many tools within futures thinking are well suited for ethnobiological research. For instance, eco-cultural mapping and seasonal calendars can serve not only as descriptive tools for past and present resource knowledge, but also as generative instruments for imagining future changes such as shifting phenologies or reconfigured land-use strategies [45, 46]. The three-horizons framework [47] enables communities and researchers to collaboratively assess which elements of their biocultural systems could be preserved, adapted, or transformed. Beyond these “close-to-home” methods, futures thinking also uses art to stimulate creativity and imagination so that futures not previously considered become visible [41, 38, 37].

Hands-on projects to co-realize imagined futures in partnership with Indigenous Peoples and local communities strengthen capacities for social-ecological resilience in the face of unexpected changes [48]. Co-designed projects aim to adapt biocultural systems by sustaining, innovating and improving specific elements, to transform these systems by creating novel pathways, or to build capacity to live with complexity, uncertainty, and change [49, 50]. Futures thinking and scenario planning can lead to identifying challenges and opportunities, as well as establishing a shared agenda. To fulfil these goals and objectives, transdisciplinary approaches are the quintessential methodology for knowledge co-production, collaborative problem-solving, and promoting transformative change [51–54]. Goal-oriented transdisciplinary approaches are fitting to ethnobiology because they are context-based and iterative (as the ethnobiological research process itself, [55]) and pluralistic (i.e., recognizing, as ethnobiology does, multiple ways of knowing, being and doing, [2]).

In the following sections, we examine three methodological approaches that hold particular promise for advancing ethnobiological research with a future-oriented perspective. Each of these approaches provides distinct entry points for engaging with communities, knowledge systems, and landscapes in ways that enable the co-creation of anticipatory insights and context-specific pathways towards biocultural resilience.

## Walking workshops

Walking workshops (i.e. community-led dialogues conducted within lived landscapes) have emerged as particularly promising methods for co-exploring future pathways [56]. Rather than confining discussions to meeting rooms or abstract scenarios, walking workshops situate dialogue in the very places where biocultural relations are enacted and sustained [57]. This embodied practice fosters deep engagement with land and seascapes as both archives of memory and spaces of aspiration. As such, they create conditions for intergenerational learning, where knowledge is not only transmitted, but also reinterpreted in the light of emerging challenges and opportunities [58, 56].

Importantly, these participatory engagements connect directly with the aspirations, needs, and rights of Indigenous Peoples, while fostering solutions to local challenges in ways that are culturally meaningful and grounded in place [59]. Unlike more formal consultation settings, being immersed in the land enables participants to reflect together, strengthening mutual understanding and opening spaces for agency, and transformative change [60], [61]. As Tengö et al. [56] emphasize, walking together on the land fosters a holistic understanding of both material and immaterial dimensions of biocultural relationships, while affirming Indigenous worldviews and collective visioning. In doing so, walking workshops can serve as laboratories of anticipation, where futures are not only discussed, but actively co-envisioned through lived encounters with place.

### Biocultural indicators

Any transdisciplinary project stems from a common understanding of a by-definition diverse team [52]. Prior to action and monitoring, establishing what success means is necessary. This can be achieved through futures thinking and scenario planning approaches. Key concepts such as sustainability or resilience often carry different meanings across cultural contexts, disciplinary fields and professional sectors [62, 63]. Yet, tools for monitoring and evaluating the outcomes of adaptive, transformative, and capacity-building initiatives are frequently rooted in standardized interpretations of these terms [52].

Among the most context-sensitive tools available are biocultural indicators, designed to capture both ecological and sociocultural dimensions in ways that are meaningful and culturally appropriate [64]. By explicitly recognizing the interconnections between human and environmental well-being, biocultural indicators provide a more holistic approach to assessment [65]. Crucially, they are developed and applied to evaluate progress towards fulfilling of the aspirations, needs, and rights of Indigenous Peoples and local communities [66], ensuring thatmonitoring efforts are aligned with local values and priorities. The effects of an initiative are shaped not only by which indicators are measured, but also by how they are measured. Because they are grounded in place-based values, knowledge, and needs, biocultural indicators reflect community governance systems (i.e., who is included, who decides for whom), worldviews (i.e., in relation to people’s responsibilities to the environment), and livelihoods (i.e., often monitored through existing social practices [66]). They often emerge from participatory processes such as scenario planning workshops, or are derived from the factors that shape envisioned futures [39, 25]. Importantly, connecting biocultural indicators with those commonly used in national and international research and policy, through iterative dialogue on their overlaps and differences [67], can help redress systemic injustices and strengthen community-driven solutions [68].

### Living laboratories

How can desirable futures, envisioned through future thinking and scenario planning methodologies, be nurtured into being? Living laboratories are collaborative platforms designed to co-create, test, and evaluate innovative solutions in real-world settings, often with the aim of moving beyond “business-as-usual” approaches [69]. While their origins lie in the development activities led by citizens, companies, non-profit organizations and other stakeholders aiming to improve their everyday lives, their applications have significantly attracted academic attention [70]. Today, living laboratories are increasingly adopted in fields seeking transformative change through collaborative innovation effort, particularly within social-ecological systems research [71], [72, 73]. They have been used to foster experimentation and learning in agriculture [74], soil health [75] medicine [76], and energy transitions [77]. In these contexts, living laboratories serve not as tools for development and commercialization of new products, but as participatory infrastructures that support inclusive knowledge co-production, place-based innovation, and systems-level change [78, 79]. Participatory monitoring and evaluation of living laboratory processes can further facilitate social learning, while strengthening and diversifying networks among participants [80].

For ethnobiology, repurposing the living laboratory model in culturally grounded, community-led ways may offer a powerful opportunity to envision and co-create biocultural futures aligned with Indigenous and local values. By fostering iterative, place-based experimentation, living laboratories provide a framework through which ethnobiologists can move beyond extractive research models and towards sustained, co-creative engagement with Indigenous and local communities [81]. These spaces are particularly well suited for supporting biocultural resilience, as they enable the development of context-specific solutions to pressing social-ecological challenges [82, [83]. In the context of a forward-looking ethnobiology, living laboratories can serve as dynamic arenas for exploring future-oriented practices, ranging from agroecological experimentation to cultural revitalization or anticipatory adaptations to global environmental change. Their transdisciplinary nature and their emphasis on long-term partnerships resonates with the ethical imperatives of promoting relational accountability with Indigenous and local knowledge holders [84, 85]. As such, living laboratories can help reorient ethnobiology towards hands-on participatory, reflexive, and futures-driven approaches.

## From vision to action: shaping ethnobiology’s futures

Ethnobiology’s strength lies in its diversity of approaches [16, 86, 87]. Embracing anticipatory methods should by no means diminish the value of past- and present-oriented work, but rather broaden our collective ability to engage with rapid social-ecological transformations [23, 88]. We believe that fostering a more inclusive methodological landscape will enrich, rather than dilute, the scope and relevance of ethnobiological inquiry.

While futures thinking and scenario planning have gained traction in global change and sustainability science [89, 90], they remain largely underutilized within the field of ethnobiology, despite innovative applications in some Indigenous contexts (e.g. [91–93]). The implementation of biocultural indicators remains far from mainstream but could find its utility niche within the context of the rapidly multiplying living laboratory organizations and projects. The broader integration of these approaches into the discipline offers significant promise, not only by fostering methodological innovation, but also by enhancing ethnobiology’s capacity to support Indigenous-led processes of transformation, resistance, and renewal. The high gain derived from infusing futures thinking within the fabric of ethnobiology lies in its ability to help the discipline realize a fundamental aspiration: to actively support the maintenance, restoration, and revitalization of ethnobiological knowledge systems [94, 17].

In recent years, numerous papers and commentaries have sought to envision the future of ethnobiology, offering valuable reflections on the directions the field should pursue and calling for its evolution towards more inclusive, decolonial, and socially engaged paradigms (e.g. [95, 87, 12]). These forward-looking contributions have outlined aspirations for ethnobiology to become more ethically reflexive and impactful in addressing contemporary social-ecological challenges [3, 8, 9]. While such visioning is essential, we argue that the time has come to complement these discursive projections with concrete, participatory action. Rather than merely anticipating what the future of ethnobiology might entail or prescribing the roles it should assume, the discipline must now engage more actively in supporting the construction of the futures it seeks to inhabit [11, 10]. This implies co-producing anticipatory research agendas with Indigenous Peoples and local communities, embedding futures-oriented methodologies into our work, and aligning scholarly practice with transformative goals. In doing so, ethnobiology can move from envisioning to enacting futures, grounded not only in ethical commitments and epistemic plurality, but also in tangible pathways for collective biocultural resilience.

## Conclusions

Forward-looking ethnobiology bridges place-based knowledge systems with anticipatory approaches, enabling communities and researchers to engage proactively with the future and navigate social-ecological change together. While ethnobiology has long embraced anticipatory and co-creative approaches, embedding Futures Studies adds value by providing a shared conceptual vocabulary and methodological repertoire that can strengthen, connect, and scale these efforts. Grounded in Indigenous knowledge, values, and practices, this approach offers structured ways to co-develop solutions to complex social-ecological challenges and to envision and enact transformative pathways. Far from departing from the discipline’s trajectory, forward-looking ethnobiology deepens its commitment to biocultural resilience, Indigenous sovereignty, and social justice. Ultimately, embracing forward-looking strategies enhances ethnobiology’s potential to foster adaptive, inclusive, and resilient futures, in which biocultural systems and the communities that sustain them can thrive amid uncertainty.

## Data Availability

No datasets were generated or analysed during the current study.
